# Commentary: Primary Emotional Systems and Personality: An Evolutionary Perspective

**DOI:** 10.3389/fpsyg.2017.02102

**Published:** 2018-01-17

**Authors:** Valentina Questa

**Affiliations:** Associazione Scuola di Psicoterapia Cognitiva, Rome, Italy

**Keywords:** emotions, personality, psychotherapy, evolution, psychopathology

In *Primary Emotional Systems and Personality* Christian Montag and Jaak Panksepp show how subjectivity emerges from basic affective processes. Their view of the affective grounds of personality has important implications for the clinic of psychiatric disorders, because emotional disfunctions could be regarded as the common trait of all different kinds of psychopathology. In Montag and Panksepp's words, “[…] emotional disturbances in childhood have the strongest effect on the development of psychopathological disorders in adults” (Montag and Panksepp, 2017).

The description of our primary emotional processes as natural kinds in terms of both their synchronic and diachronic dimensions—i.e., through both a functional description of the processes and a genealogical one—is epistemologically and substantively meaningful. From an epistemological point of view, such a description fosters a naturalized, Darwinian and interdisciplinary understanding of human nature, which is far from any kind of biological reductionism. From a substantive point of view, it affirms the nature and the functional priority of the emotional processes for the constitution of personality, because “individual differences in emotionality could represent the evolutionary oldest part of human personality” (Montag and Panksepp, 2017). A scientific psychotherapy should be based on a clinical theory built upon these premises.

Montag and Panksepp's work on the affective roots of personality provides a fruitful and solid framework for researchers on psychotherapy, a practice that calls for high ethical and scientific standards because of its role in promoting human wellbeing. However, currently it is difficult to recognize a set of core hypotheses common to psychological research, clinical theory, the analysis of psychiatric concepts, and therapeutic practices. On the contrary, it would be fruitful to find scientifically sound research programs shared by various approaches to psychotherapy within the framework of the most up-to-date cognitive science.

Montag and Panksepp underline the phylogenetic primacy of basic emotions in cognitive processes. Primary emotional systems are the primigenial mechanism responsible for the reading and evaluation of reality. These systems are homologous for at least all mammals' nervous systems and they lie at the core of both motivation and agency. The qualitative and subjective aspect of affectivity is essential to understand the basis of our development and the validity of many animal experimental models, in opposition to the skeptical or agnostic attitude of more traditional cognitive neurosciences (Panksepp, 2011).

The species gene pool filtered by the peculiar affective first-person experience causes a set of readings and evaluations that constitute the earliest kind of learning. These would shape the neocortical complex cognitive faculties and the individual character and personality, through particular adaptive pathways that are mainly relational. In all of his previous work Panksepp stresses how this fundamental qualitative trait of our feelings shapes us and builds our experiences (Panksepp and Biven, 2012). This trait is the core of our phenomenal awareness. It is a datum that can be scientifically investigated from a third person perspective, like other facts.

Data about animal conditioning, for example, can be explained only by setting out from the premise that pleasurable or unpleasurable feelings drive the animal to reach or avoid some stimuli. Motivation to learn, like the whole set of preferences expressed by a lab animal, can be understood only from the affective point of view (Panksepp, 2011). Without the primary subjective-affective component, innate knowledges about the properties of objects (folk physics) cannot give value to anything. By stressing the primary feature of emotional processes, affective neurosciences contribute to eradicating the rationalistic, logocentric, and discontinuist bias about human nature (Davidson, 1982). They provide evidence against any cognitivist theory of emotions grounded in such a bias (Nussbaum, 2001).

Any research on human nature must be based on the recognition that we share most of our gene pool, capacities and function with all the rest of animal kingdom, from which we differ, like any other species, because of some specific traits. This common heritage is the starting point to understand our distinctive features, whose precursors can be traced back along the paths of our phylogeny and recognized in non-human species.

The linguistic translation of our knowledge is a late and derived process, from both a phylogenetic and ontogenetic point of view. Early emotional memories of relationships are codified into psychobiological circuits of the right hemisphere, which is dominant during the first years of development (Shore, 2003). Once stored, these memories will constitute the computational structures which Bowlby refers to as “internal working models” (Bowlby, 1988). These are stable ways of feeling in relation to others and they adaptively give birth to stable character traits along a continuum from healthy to pathological ones.

The therapeutic relationship should modify those interactional patterns acquired because of their adaptive value in a dysfunctional environment during the development. It should operate through the same early ways of learning in order to disconfirm those patterns in the here and now of therapeutic experience. This happens by means of relational, implicit and emotional communication between therapist and patient (Safran and Muran, 2000).

The patient's awareness of the harmfulness of her patterns is not enough to modify them, insofar as they are still perceived as safe strategies capable of promptly reducing anxious states, compared to unknown pathways, that are not recommended from an evolutionary standpoint (“better safe than sorry”). Motivation to change is not produced by a cognitive understanding of reasons, advantages or the potentially endless pros and cons. It is fostered by the capacity to feel safe in the immediacy of the affective experiencing of positive emotions. Therefore, the cognitivist view that changing dysfunctional beliefs is the purpose of good psychotherapy (Beck, 1976; Pretzer and Beck, 2005) should be regarded as simplistic. Such a change is at best an epiphenomenal effect that acts as a feedback on the deepest emotional and motivational processes.

Reason, which is intrinsically inert, is and must be slave of the passions, as stated in the eighteenth century by Hume (2000), a philosopher often quoted by Panksepp. In philosophy, Hume carried out a paradigm reversal through the recognition of the primacy of emotions over reason and their value for knowledge. Panksepp produces a similar reversal in neuroscience, offering a rich and fruitful framework for present and future research.

## Author contributions

The author confirms being the sole contributor of this work and approved it for publication.

### Conflict of interest statement

The author declares that the research was conducted in the absence of any commercial or financial relationships that could be construed as a potential conflict of interest.
